# Supplementary material for: Evaluating variant pathogenicity prediction tools to establish African inclusive guidelines for germline genetic testing
Source: Commun Med (Lond). 2025 May 6;5:157. doi: 10.1038/s43856-025-00883-x (PMC12056225; doi:10.1038/s43856-025-00883-x)
Supplement: Supplementary file 2 — Supplementary Information [file 43856_2025_883_MOESM2_ESM.pdf]

Evaluating variant pathogenicity prediction tools to establish African inclusive guidelines for germline genetic testing

Kangping Zhou, Kazzem Gheybi, Pamela X.Y. Soh & Vanessa M. Hayes

SUPPLEMENTARY DOCUMENT

Supplementary tables

Supplementary Table 1. Performance of 54 VPPTs for ancestry-specific African (AFR) *vs* European (EUR) advanced prostate cancer with the merged benchmark datasets classified by ClinVar and InterVar (ACMG-AMP guidelines) and ordered in descending order of sensitivity (Sen) for African ancestral patients.

Supplementary Table 2. Raw number of positive pathogenic and negative benign variants from ancestry-specific African (AFR) *vs* European (EUR) advanced prostate cancer men predicted by 54 VPPTs with the merged benchmark datasets classified by ClinVar and InterVar (ACMG-AMP guidelines).

Supplementary Table 3. Performance of 54 VPPTs for ancestry-specific African (AFR) *vs* European (EUR) advanced prostate cancer with the benchmark datasets classified by ClinVar only and ordered in descending order of sensitivity (Sen) for African ancestral patients.

Supplementary Table 4. Raw number of positive pathogenic and negative benign variants from ancestry-specific African (AFR) *vs* European (EUR) advanced prostate cancer men predicted by 54 VPPTs with the benchmark datasets classified by ClinVar.

Supplementary Table 5. Raw number of positive pathogenic and negative benign variants from ancestry-specific African (AFR) *vs* European (EUR) advanced prostate cancer men predicted by 54 VPPTs with the benchmark datasets classified by InterVar (ACMG-AMP guidelines).

Supplementary Figures

Supplementary Figure 1 | Sensitivity across 54 VPPTs for ancestry-specific African (AFR) *vs* European (EUR) benchmark datasets classified by the ACMG-AMP guidelines using InterVar. Ranked from highest to lowest (left to right) sensitivity (sen) for African (AFR, orange), with further comparison with European (EUR, blue) patient-matched benchmark data.

Supplementary Table 1. Performance of 54 VPPTs for ancestry-specific African (AFR) *vs* European (EUR) advanced prostate cancer with the merged benchmark datasets classified by ClinVar and InterVar (ACMG-AMP guidelines) and ordered in descending order of sensitivity (Sen) for African ancestral patients.

Tool	Sen AFR	Sen EUR	Spec AFR	Spec EUR	FPR AFR	FPR EUR	FNR AFR	FNR EUR	MCC AFR	MCC EUR
M-CAP	0.98	0.96	0.59	0.59	0.41	0.41	0.02	0.04	0.21	0.22
GERP-NR	0.92	0.94	0.19	0.20	0.81	0.80	0.08	0.06	0.04	0.05
MetaSVM	0.92	0.74	0.93	0.93	0.07	0.07	0.08	0.26	0.38	0.28
phastCons470way-mammalian	0.92	0.90	0.55	0.50	0.45	0.50	0.08	0.10	0.16	0.12
CADD	0.91	0.97	0.71	0.66	0.29	0.34	0.09	0.03	0.22	0.19
GenoCanyon	0.90	0.90	0.27	0.27	0.73	0.73	0.10	0.10	0.06	0.06
MutationTaster	0.90	0.90	0.64	0.61	0.36	0.39	0.10	0.10	0.16	0.13
fathmm-MKL	0.88	0.88	0.59	0.55	0.41	0.45	0.12	0.12	0.16	0.13
MetaLR	0.88	0.72	0.91	0.92	0.09	0.08	0.12	0.28	0.33	0.26
phastCons100way-vertebrate	0.88	0.91	0.55	0.51	0.45	0.49	0.12	0.09	0.14	0.12
phyloP470way-mammalian	0.87	0.89	0.57	0.53	0.43	0.47	0.13	0.11	0.15	0.12
Eigen-raw	0.86	0.89	0.77	0.72	0.23	0.28	0.14	0.11	0.24	0.19
Eigen-PC	0.82	0.87	0.75	0.70	0.25	0.30	0.18	0.13	0.22	0.18
LINSIGHT	0.81	0.90	0.67	0.73	0.33	0.27	0.19	0.10	0.46	0.52
phyloP100way-vertebrate	0.80	0.83	0.66	0.61	0.34	0.39	0.20	0.17	0.16	0.13
bStatistic	0.79	0.71	0.20	0.20	0.80	0.80	0.21	0.29	-0.01	-0.03
DANN	0.79	0.76	0.71	0.67	0.29	0.33	0.21	0.24	0.18	0.13
phastCons17way-primate	0.79	0.77	0.52	0.50	0.48	0.50	0.21	0.23	0.10	0.08
BayesDel-noAF	0.78	0.87	0.93	0.91	0.07	0.09	0.22	0.13	0.41	0.36
LRT	0.77	0.75	0.74	0.70	0.26	0.30	0.23	0.25	0.18	0.14
Polyphen2-HDIV	0.77	0.87	0.64	0.61	0.36	0.39	0.23	0.13	0.11	0.11
SIFT	0.77	0.80	0.65	0.64	0.35	0.36	0.23	0.20	0.11	0.10
MVP	0.75	0.74	0.85	0.84	0.15	0.16	0.25	0.26	0.21	0.21
LIST.S2	0.74	0.75	0.77	0.74	0.23	0.26	0.26	0.25	0.15	0.12
SIFT4G	0.74	0.77	0.74	0.71	0.26	0.29	0.26	0.23	0.14	0.12
MutationAssessor	0.72	0.87	0.74	0.71	0.26	0.29	0.28	0.13	0.14	0.15
GERP-RS	0.71	0.76	0.74	0.70	0.26	0.30	0.29	0.24	0.17	0.14
REVEL	0.70	0.76	0.95	0.93	0.05	0.07	0.30	0.24	0.35	0.30
BayesDel-addAF	0.69	0.70	0.99	0.99	0.01	0.01	0.31	0.30	0.65	0.59
ESM1b	0.68	0.71	0.83	0.80	0.17	0.20	0.32	0.29	0.17	0.15
SiPhy	0.68	0.75	0.76	0.73	0.24	0.27	0.32	0.25	0.17	0.16
ClinPred	0.67	0.48	0.99	0.99	0.01	0.01	0.33	0.52	0.57	0.47
Polyphen2-HVAR	0.67	0.80	0.76	0.73	0.24	0.27	0.33	0.20	0.13	0.14
FATHMM	0.66	0.65	0.82	0.84	0.18	0.16	0.34	0.35	0.16	0.15
PROVEAN	0.66	0.76	0.81	0.77	0.19	0.23	0.34	0.24	0.15	0.14
MutPred	0.63	0.74	0.78	0.78	0.22	0.22	0.38	0.26	0.20	0.18
fathmm-XF	0.62	0.59	0.80	0.77	0.20	0.23	0.38	0.41	0.15	0.12
VEST4	0.62	0.74	0.85	0.85	0.15	0.15	0.38	0.26	0.19	0.21
VARITY-R	0.61	0.70	0.92	0.90	0.08	0.10	0.39	0.30	0.25	0.22
VARITY-R-LOO	0.61	0.70	0.92	0.90	0.08	0.10	0.39	0.30	0.24	0.23
DEOGEN2	0.60	0.59	0.93	0.93	0.07	0.07	0.40	0.41	0.25	0.22
MetaRNN	0.58	0.43	1.00	1.00	0.00	0.00	0.42	0.57	0.62	0.49
hESC-fitCons	0.55	0.59	0.34	0.36	0.66	0.64	0.45	0.41	-0.04	-0.02
EVE	0.54	0.59	0.81	0.81	0.19	0.19	0.46	0.41	0.18	0.19
integrated-fitCons	0.54	0.53	0.38	0.40	0.62	0.60	0.46	0.47	-0.03	-0.02
HUVEC-fitCons	0.53	0.48	0.39	0.40	0.61	0.60	0.47	0.52	-0.03	-0.04
gMVP	0.50	0.61	0.94	0.92	0.06	0.08	0.50	0.39	0.23	0.22
VARITY-ER	0.50	0.65	0.94	0.92	0.06	0.08	0.50	0.35	0.23	0.23
VARITY-ER-LOO	0.49	0.63	0.94	0.92	0.06	0.08	0.51	0.37	0.22	0.22
AlphaMissense	0.43	0.57	0.96	0.94	0.04	0.06	0.57	0.43	0.22	0.20
GM12878-fitCons	0.39	0.45	0.54	0.54	0.46	0.46	0.61	0.55	-0.03	0.00
PrimateAI	0.20	0.15	0.97	0.97	0.03	0.03	0.80	0.85	0.13	0.08
MPC	0.03	0.04	1.00	0.99	0.00	0.01	0.97	0.96	0.04	0.05
phyloP17way-primate	0.00	0.00	1.00	1.00	0.00	0.00	1.00	1.00	0.00	0.00
Mean	0.68	0.70	0.74	0.72	0.26	0.28	0.32	0.30	0.19	0.17
p-value	*2.62E-02*		*2.92E-06*		*2.92E-06*		*2.62E-02*		*8.57E-06*	

Abbreviations: Acc, accuracy; AFR, African ancestry; EUR, European ancestry; FNR, false negative rate; FPR, false positive rate; MCC, Matthew's correlation coefficient; Sen, sensitivity; Spec, specificity.

Supplementary Table 2. Raw number of positive pathogenic and negative benign variants from ancestry-specific African (AFR) *vs* European (EUR) advanced prostate cancer men predicted by 54 VPPTs with the merged benchmark datasets classified by ClinVar and InterVar (ACMG-AMP guidelines).

Tool	**TP AFR**	TP EUR	**FP AFR**	FP EUR	**TN AFR**	TN EUR	**FN AFR**	FN EUR
phastCons470way-mammalian	142	167	2415	4248	2952	4295	12	19
GERP-NR	141	174	4345	6842	1013	1697	13	12
CADD	140	180	1559	2949	3821	5622	14	6
GenoCanyon	138	168	3900	6216	1475	2345	16	18
phastCons100way-vertebrate	136	169	2395	4193	2985	4378	18	17
fathmm-MKL	135	164	2183	3836	3192	4725	19	22
Eigen-raw	131	159	1195	2293	3961	5917	21	20
Eigen-PC	125	156	1290	2470	3866	5740	27	23
phyloP100way-vertebrate	123	155	1838	3341	3542	5230	31	31
phyloP470way-mammalian	122	154	2061	3586	2784	4028	19	19
DANN	121	141	1532	2833	3843	5728	33	45
phastCons17way-primate	121	144	2560	4321	2820	4250	33	42
BayesDel-noAF	120	161	364	767	4980	7726	34	25
bStatistic	120	130	4257	6722	1048	1718	32	54
GERP-RS	110	142	1405	2597	3953	5942	44	44
BayesDel-addAF	106	130	63	120	5281	8373	48	56
MutationTaster	106	111	1913	3240	3376	5115	12	12
SiPhy	104	140	1284	2258	4027	6206	49	46
hESC-fitCons	84	109	3486	5383	1776	2982	69	75
LRT	83	100	1019	1882	2930	4407	25	33
integrated-fitCons	82	97	3244	5030	2018	3335	71	87
HUVEC-fitCons	81	88	3214	5007	2048	3358	72	96
M-CAP	80	94	887	991	1296	1452	2	4
MetaSVM	77	78	360	562	4852	7735	7	28
VEST4	77	113	783	1282	4442	7040	47	40
fathmm-XF	75	91	1043	1874	4095	6255	46	62
MetaLR	74	76	467	641	4745	7656	10	30
MVP	64	78	750	856	4356	4344	21	28
SIFT	64	82	1725	2898	3270	5046	19	20
Polyphen2-HDIV	63	88	1638	2900	2938	4446	19	13
SIFT-4G	62	83	1330	2345	3712	5630	22	25
LIST-S2	61	77	1144	2109	3931	5957	21	26
GM12878-fitCons	59	82	2438	3815	2824	4550	94	102
ClinPred	58	51	59	57	5146	8235	29	56
MutationAssessor	58	82	1123	2013	3182	4845	23	12
REVEL	58	80	247	528	4818	7540	25	25
ESM1b	57	74	827	1529	3988	6172	27	30
Polyphen2-HVAR	55	81	1119	2007	3457	5339	27	20
PROVEAN	55	79	972	1813	4060	6194	28	25
FATHMM	54	66	886	1251	4004	6496	28	35
DEOGEN2	50	61	336	563	4410	7023	34	42
MetaRNN	50	47	24	34	5298	8426	36	63
VARITY-R	50	69	359	730	4193	6557	32	29
VARITY-R-LOO	50	69	384	705	4168	6582	32	29
gMVP	41	65	284	586	4555	7129	41	42
VARITY-ER	41	64	272	585	4280	6702	41	34
VARITY-ER-LOO	40	62	274	590	4278	6697	42	36
AlphaMissense	32	47	210	492	4785	7337	43	36
EVE	29	35	209	297	918	1230	25	24
LINSIGHT	25	28	18	33	36	88	6	3
MutPred	25	20	190	255	680	892	15	7
PrimateAI	17	16	139	233	4655	7372	67	89
MPC	2	4	20	43	4383	6993	77	98
phyloP17way-primate	0	0	0	0	5380	8571	154	186
**Mean**	**77.30**	95.94	**1259.98**	2125.02	**3496.78**	5363.85	**34.30**	38.91
p-value	*6.70E-16*		*4.31E-12*		*1.55E-22*		*2.54E-03*	

Abbreviations: AFR, African ancestry; EUR, European ancestry; FN, false negatives; FP, false positives; TN, true negatives; TP, true positives.

Supplementary Table 3. Performance of 54 VPPTs for ancestry-specific African (AFR) *vs* European (EUR) advanced prostate cancer with the benchmark datasets classified by ClinVar and ordered in descending order of sensitivity (Sen) for African ancestral patients.

Tool	**Sen AFR**	Sen EUR	**Spec AFR**	Spec EUR	**FPR AFR**	FPR EUR	**FNR AFR**	FNR EUR	**MCC AFR**	MCC EUR
phastCons470way-mammalian	0.97	0.94	0.42	0.35	0.58	0.65	0.03	0.06	0.08	0.08
M.CAP	0.95	0.96	0.56	0.57	0.44	0.43	0.05	0.04	0.11	0.18
CADD	0.95	0.99	0.60	0.52	0.40	0.48	0.05	0.01	0.11	0.13
GERP-NR	0.95	0.97	0.11	0.11	0.89	0.89	0.05	0.03	0.02	0.03
LIST.S2	0.94	0.73	0.70	0.65	0.30	0.35	0.06	0.27	0.10	0.08
BayesDel-noAF	0.92	0.89	0.91	0.87	0.09	0.13	0.08	0.11	0.28	0.28
fathmm-MKL	0.92	0.93	0.46	0.40	0.54	0.60	0.08	0.07	0.08	0.09
GenoCanyon	0.92	0.96	0.23	0.21	0.77	0.79	0.08	0.04	0.04	0.05
phastCons100way-vertebrate	0.92	0.96	0.43	0.37	0.57	0.63	0.08	0.04	0.07	0.08
Eigen-raw	0.92	0.90	0.68	0.60	0.32	0.40	0.08	0.10	0.13	0.13
REVEL	0.90	0.80	0.93	0.91	0.07	0.09	0.10	0.20	0.25	0.26
SIFT	0.90	0.83	0.64	0.60	0.36	0.40	0.10	0.17	0.09	0.10
Polyphen2-HDIV	0.89	0.90	0.60	0.54	0.40	0.46	0.11	0.10	0.08	0.10
DANN	0.89	0.81	0.63	0.57	0.37	0.43	0.11	0.19	0.11	0.10
phyloP470way-mammalian	0.89	0.93	0.45	0.38	0.55	0.62	0.11	0.07	0.07	0.08
MutationTaster	0.87	0.93	0.54	0.48	0.46	0.52	0.13	0.07	0.07	0.08
MVP	0.85	0.73	0.81	0.80	0.19	0.20	0.15	0.27	0.13	0.16
PROVEAN	0.85	0.77	0.78	0.74	0.22	0.26	0.15	0.23	0.12	0.12
ESM1b	0.84	0.72	0.80	0.76	0.20	0.24	0.16	0.28	0.12	0.12
MutationAssessor	0.84	0.85	0.70	0.65	0.30	0.35	0.16	0.15	0.09	0.12
VARITY-R	0.84	0.80	0.90	0.88	0.10	0.12	0.16	0.20	0.19	0.22
VARITY-R-LOO	0.84	0.80	0.89	0.87	0.11	0.13	0.16	0.20	0.18	0.22
LRT	0.84	0.82	0.68	0.62	0.32	0.38	0.16	0.18	0.12	0.11
BayesDel-addAF	0.84	0.69	0.98	0.98	0.02	0.02	0.16	0.31	0.54	0.50
phastCons17way-primate	0.84	0.81	0.44	0.39	0.56	0.61	0.16	0.19	0.06	0.05
phyloP100way-vertebrate	0.84	0.88	0.53	0.46	0.47	0.54	0.16	0.12	0.08	0.09
VEST4	0.81	0.84	0.80	0.79	0.20	0.21	0.19	0.16	0.14	0.18
bStatistic	0.81	0.79	0.20	0.21	0.80	0.79	0.19	0.21	0.00	0.00
Eigen-PC	0.81	0.92	0.64	0.57	0.36	0.43	0.19	0.08	0.10	0.13
gMVP	0.80	0.71	0.93	0.90	0.07	0.10	0.20	0.29	0.21	0.22
LINSIGHT	0.80	0.91	0.57	0.70	0.43	0.30	0.20	0.09	0.24	0.40
MetaSVM	0.80	0.66	0.90	0.90	0.10	0.10	0.20	0.34	0.18	0.20
SIFT4G	0.80	0.82	0.71	0.67	0.29	0.33	0.20	0.18	0.09	0.11
DEOGEN2	0.79	0.60	0.91	0.90	0.09	0.10	0.21	0.40	0.18	0.17
Polyphen2-HVAR	0.79	0.84	0.71	0.67	0.29	0.33	0.21	0.16	0.09	0.12
EVE	0.79	0.70	0.80	0.80	0.20	0.20	0.21	0.30	0.18	0.20
SiPhy	0.78	0.79	0.67	0.63	0.33	0.37	0.22	0.21	0.10	0.11
FATHMM	0.75	0.62	0.79	0.81	0.21	0.19	0.25	0.38	0.10	0.12
MutPred	0.75	0.83	0.74	0.75	0.26	0.25	0.25	0.17	0.15	0.18
VARITY-ER	0.74	0.73	0.92	0.90	0.08	0.10	0.26	0.27	0.19	0.22
ClinPred	0.73	0.50	1.00	0.99	0.00	0.01	0.27	0.50	0.60	0.49
MetaLR	0.70	0.65	0.88	0.89	0.12	0.11	0.30	0.35	0.14	0.18
MetaRNN	0.70	0.49	1.00	1.00	0.00	0.00	0.30	0.51	0.61	0.51
VARITY-ER-LOO	0.68	0.75	0.92	0.90	0.08	0.10	0.32	0.25	0.17	0.23
fathmm-XF	0.68	0.68	0.71	0.67	0.29	0.33	0.32	0.32	0.08	0.09
GERP-RS	0.65	0.81	0.62	0.56	0.38	0.44	0.35	0.19	0.06	0.09
AlphaMissense	0.65	0.75	0.95	0.92	0.05	0.08	0.35	0.25	0.19	0.22
hESC-fitCons	0.61	0.65	0.30	0.31	0.70	0.69	0.39	0.35	-0.02	-0.01
HUVEC-fitCons	0.58	0.56	0.36	0.36	0.64	0.64	0.42	0.44	-0.01	-0.02
integrated-fitCons	0.58	0.62	0.33	0.34	0.67	0.66	0.42	0.38	-0.02	-0.01
GM12878-fitCons	0.47	0.54	0.51	0.51	0.49	0.49	0.53	0.46	0.00	0.01
PrimateAI	0.25	0.08	0.97	0.96	0.03	0.04	0.75	0.92	0.10	0.03
MPC	0.00	0.03	1.00	0.99	0.00	0.01	1.00	0.97	-0.01	0.04
phyloP17way-primate	0.00	0.00	1.00	1.00	0.00	0.00	1.00	1.00	0.00	0.00
**Mean**	0.77	0.74	0.69	0.66	0.31	0.34	0.23	0.26	0.13	0.14
***p*-value**	*6.28E-02*		*6.53E-06*		*6.53E-06*		*6.28E-02*		*3.19E-02*	

Abbreviations: Acc, accuracy; AFR, African ancestry; EUR, European ancestry; FNR, false negative rate; FPR, false positive rate; MCC, Matthew's correlation coefficient; Sen, sensitivity; Spec, specificity.

Supplementary Table 4. Raw number of positive pathogenic and negative benign variants from ancestry-specific African (AFR) *vs* European (EUR) advanced prostate cancer men predicted by 54 VPPTs with the benchmark datasets classified by ClinVar.

Tool	**TP AFR**	TP EUR	**FP AFR**	FP EUR	**TN AFR**	TN EUR	**FN AFR**	FN EUR
phastCons470way-mammalian	36	84	2019	3558	1449	1889	1	5
CADD	35	88	1385	2608	2088	2852	2	1
GERP-NR	35	86	3072	4819	394	626	2	3
BayesDel-noAF	34	79	325	689	3125	4715	3	10
fathmm-MKL	34	83	1859	3283	1609	2167	3	6
GenoCanyon	34	85	2683	4282	785	1168	3	4
phastCons100way-vertebrate	34	85	1974	3456	1499	2004	3	4
DANN	33	72	1277	2336	2191	3114	4	17
Eigen-raw	33	79	1081	2069	2247	3146	3	9
phyloP470way-mammalian	32	79	1768	3084	1439	1885	4	6
BayesDel-addAF	31	61	55	99	3395	5305	6	28
phastCons17way-primate	31	72	1945	3314	1528	2146	6	17
phyloP100way-vertebrate	31	78	1628	2933	1845	2527	6	11
bStatistic	29	70	2746	4249	694	1145	7	19
Eigen-PC	29	81	1189	2250	2139	2965	7	7
SiPhy	29	70	1135	2003	2317	3425	8	19
LRT	26	58	895	1663	1896	2698	5	13
VEST4	26	65	698	1147	2715	4201	6	12
GERP-RS	24	72	1320	2408	2146	3037	13	17
hESC-fitCons	22	58	2376	3699	1014	1629	14	31
fathmm-XF	21	53	950	1722	2370	3458	10	25
HUVEC-fitCons	21	50	2183	3415	1207	1913	15	39
integrated-fitCons	21	55	2260	3512	1130	1816	15	34
MutationTaster	20	50	1586	2764	1827	2548	3	4
M.CAP	18	53	678	809	854	1081	1	2
REVEL	18	49	223	464	3073	4671	2	12
SIFT	18	50	1183	2010	2082	3069	2	10
GM12878-fitCons	17	48	1669	2612	1721	2716	19	41
LIST.S2	17	43	988	1826	2301	3333	1	16
MVP	17	44	639	749	2703	3085	3	16
Polyphen2-HDIV	17	52	1234	2198	1826	2582	2	6
PROVEAN	17	46	715	1339	2569	3765	3	14
ClinPred	16	31	16	32	3347	5222	6	31
ESM1b	16	44	658	1216	2553	3800	3	17
gMVP	16	45	241	503	2973	4527	4	18
MetaSVM	16	41	331	525	3032	4724	4	21
MutationAssessor	16	47	877	1574	2029	2981	3	8
SIFT4G	16	51	934	1663	2343	3437	4	11
VARITY-R	16	48	311	588	2756	4194	3	12
VARITY-R-LOO	16	48	333	611	2734	4171	3	12
DEOGEN2	15	36	290	504	2872	4407	4	24
FATHMM	15	36	674	947	2565	4058	5	22
Polyphen2-HVAR	15	49	888	1595	2172	3185	4	9
MetaLR	14	40	399	568	2964	4681	6	22
MetaRNN	14	31	12	26	3419	5344	6	32
VARITY-ER	14	44	233	502	2834	4280	5	16
VARITY-ER-LOO	13	45	235	498	2832	4284	6	15
AlphaMissense	11	33	173	398	3021	4505	6	11
EVE	11	26	179	268	732	1057	3	11
MutPred	6	10	111	159	313	484	2	2
PrimateAI	5	5	99	178	3140	4870	15	56
LINSIGHT	4	10	16	27	21	64	1	1
MPC	0	2	13	32	2956	4591	20	57
phyloP17way-primate	0	0	0	0	3473	5460	37	89
**Mean**	**20.46**	52.22	**977.06**	1662.65	**2134.43**	3166.80	**6.15**	17.69
p-value	*2.06E-23*		*4.97E-13*		*4.10E-20*		*1.18E-10*	

Abbreviations: AFR, African ancestry; EUR, European ancestry; FN, false negatives; FP, false positives; TN, true negatives; TP, true positives.

Supplementary Table 5. Raw number of positive pathogenic and negative benign variants from ancestry-specific African (AFR) *vs* European (EUR) advanced prostate cancer men predicted by 54 VPPTs with the benchmark datasets classified by InterVar (ACMG-AMP guidelines).

Tool	**TP AFR**	TP EUR	**FP AFR**	FP EUR	**TN AFR**	TN EUR	**FN AFR**	FN EUR
GERP-NR	121	131	2938	4713	813	1435	12	10
phastCons470way-mammalian	121	125	1369	2487	2385	3656	12	16
CADD	120	136	797	1545	2968	4622	13	5
GenoCanyon	119	124	2635	4258	1130	1909	14	17
phastCons100way-vertebrate	116	126	1388	2486	2377	3681	17	15
fathmm-MKL	115	123	1220	2178	2545	3989	18	18
Eigen-raw	114	124	605	1186	3004	4754	18	11
Eigen-PC	109	118	638	1268	2971	4672	23	17
phyloP100way-vertebrate	105	115	947	1831	2818	4336	28	26
phastCons17way-primate	104	111	1623	2772	2142	3395	29	30
phyloP470way-mammalian	103	113	1110	1996	2252	3415	18	16
bStatistic	102	92	2978	4842	728	1230	29	47
DANN	102	107	858	1623	2907	4544	31	34
BayesDel-noAF	101	125	157	362	3588	5767	32	16
GERP-RS	97	106	648	1310	3103	4838	36	35
MutationTaster	94	86	1059	1848	2656	4192	10	7
BayesDel-addAF	90	110	19	49	3726	6080	43	31
SiPhy	89	107	658	1222	3051	4862	43	34
hESC-fitCons	71	77	2391	3747	1287	2268	62	62
HUVEC-fitCons	71	63	2224	3538	1454	2477	62	76
integrated-fitCons	71	69	2214	3471	1464	2544	62	70
LRT	67	66	529	1008	2152	3378	21	26
M-CAP	64	62	375	385	710	730	1	2
MetaSVM	63	57	161	279	3510	5765	3	7
MetaLR	62	56	236	336	3435	5708	4	8
VEST4	61	80	384	611	3266	5386	43	29
fathmm-XF	59	59	504	966	3083	4906	43	50
GM12878-fitCons	51	61	1686	2661	1992	3354	82	78
MVP	49	53	429	415	3169	2840	18	13
Polyphen2-HDIV	48	52	1026	1810	2162	3481	17	9
SIFT	48	51	1137	1922	2369	3837	17	11
SIFT-4G	48	50	857	1550	2685	4206	18	16
ClinPred	46	38	34	20	3629	6012	23	27
LIST-S2	46	50	614	1171	2960	4666	20	13
MutationAssessor	44	48	700	1226	2292	3665	20	7
ESM1b	43	45	479	895	2876	4636	24	17
Polyphen2-HVAR	42	47	675	1200	2513	4091	23	14
REVEL	42	50	121	273	3441	5587	23	14
FATHMM	41	47	567	856	2846	4754	23	15
PROVEAN	40	51	588	1124	2947	4684	25	13
MetaRNN	38	33	8	6	3728	6109	30	34
DEOGEN2	36	40	178	322	3117	5105	31	21
VARITY-R	36	39	185	379	2978	4819	29	18
VARITY-R-LOO	36	39	206	399	2957	4799	29	18
VARITY-ER	29	35	138	306	3025	4892	36	22
VARITY-ER-LOO	29	37	140	296	3023	4902	36	20
gMVP	26	36	149	340	3222	5184	38	28
LINSIGHT	24	27	10	11	23	43	6	3
AlphaMissense	22	29	111	269	3426	5442	37	26
EVE	20	22	121	190	628	888	22	14
MutPred	19	16	134	167	508	658	13	6
PrimateAI	13	13	76	131	3255	5316	53	51
MPC	2	2	14	22	3066	5056	59	61
phyloP17way-primate	0	0	0	0	3765	6167	133	141
**Mean**	**63.50**	68.13	**760.15**	1301.44	**2557.91**	4069.11	**29.85**	26.39
p-value	*1.83E-05*		*9.65E-11*		*3.70E-23*		*1.25E-03*	

Abbreviations: AFR, African ancestry; EUR, European ancestry; FN, false negatives; FP, false positives; TN, true negatives; TP, true positives.

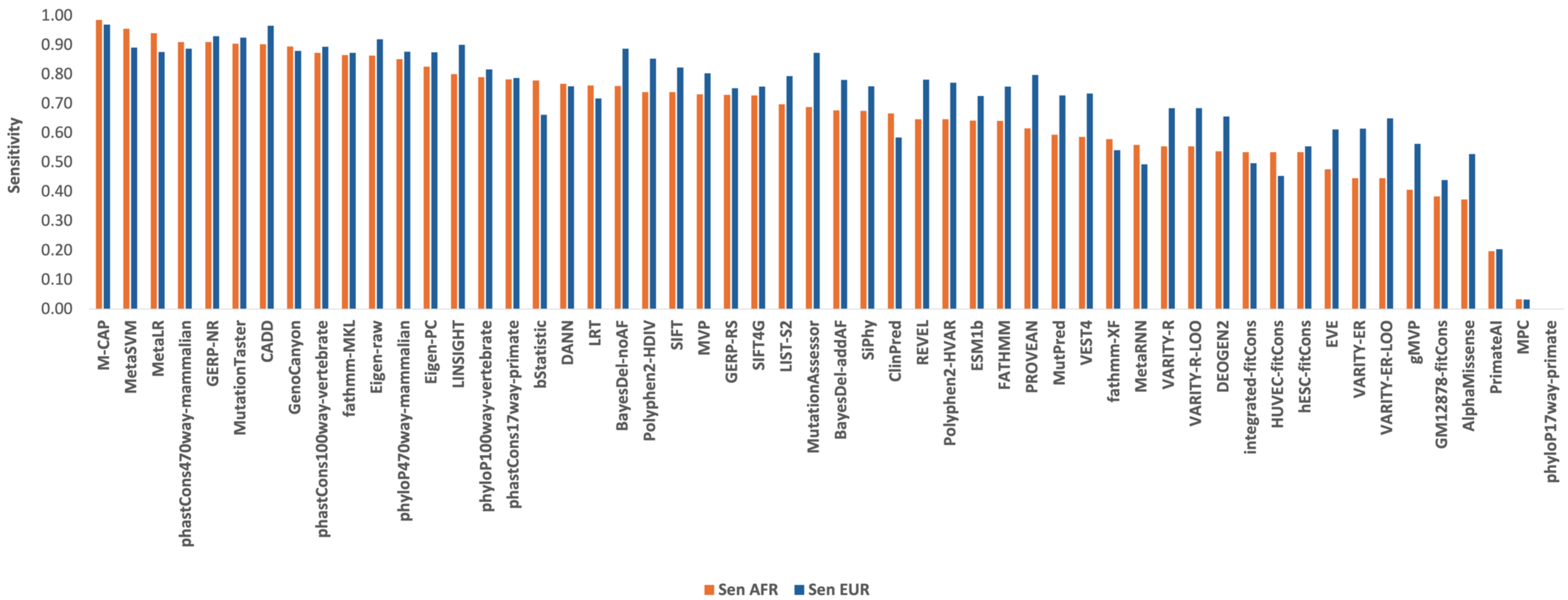

Supplementary Figure 1 | Sensitivity across 54 VPPTs for ancestry-specific African (AFR) *vs* European (EUR) benchmark datasets classified by the ACMG-AMP guidelines using InterVar. Ranked from highest to lowest (left to right) sensitivity (sen) for African (AFR, orange), with further comparison with European (EUR, blue) patient-matched benchmark data.
